# Drug Trial Decision‐Making Among Patients with Mild Cognitive Impairment and their Study Partners

**DOI:** 10.1002/alz.091104

**Published:** 2025-01-09

**Authors:** Chelsea G Cox, Christian R Salazar, Daniel L Gillen, Joshua D Grill

**Affiliations:** ^1^ University of Michigan School of Public Health, Ann Arbor, MI USA; ^2^ Institute for Memory Impairments and Neurological Disorders, University of California, Irvine, Irvine, CA USA

## Abstract

**Background:**

Prodromal Alzheimer’s disease (AD) clinical trials of candidate treatments enroll individuals with mild cognitive impairment (MCI) and biomarker evidence of AD. These trials require co‐enrollment with a study partner and complex decision‐making, weighing potential risks and benefits of participation. Some patients with MCI lack capacity to provide trial informed consent.

**Method:**

We performed structured interviews with patients with MCI and their study partners. We presented a description of a hypothetical prodromal AD drug trial, followed by questionnaires to assess (a) involvement of the study partner in trial decisions, (b) patient capacity to consent, and (c) patient performance in health numeracy. We assessed level of agreement among dyads using the weighted kappa statistic and examined the frequency with which patients demonstrated impaired health numeracy and lack of capacity to provide consent.

**Result:**

Among 65 patients with MCI, most were male (66%) and non‐Hispanic White (88%) with a mean (SD) age of 74.9 (8.4) years. Among 57 study partners, most were spouses (83%), female (74%), and non‐Hispanic White (91%) with a mean (SD) age of 70.3 (13.6). Half of patients (51%) and 72% of study partners reported being very or extremely likely to participate in the trial. Most patients (66%) and study partners (72%) stated that they would make the decision to enroll in the trial in partnership, while 28% of patients said they would make the decision on their own with input from their partner. Half of dyads (50%) agreed the decision would be made in partnership, but we found limited dyadic agreement overall (kappa = 0.187, 95% CI: ‐0.057‐0.431). Thirty‐eight percent of patients lacked capacity to consent. Patient health numeracy scores ranged from 3‐12 with a mean (SD) of 8.6 (2.5); 38% demonstrated impaired health numeracy. Sixteen patients (25%) demonstrated both a lack of capacity and impaired health numeracy, among whom seven patients reported they would make trial decisions on their own with input from a partner.

**Conclusion:**

Some patients with MCI may be at risk for decision errors in the informed consent process for a prodromal AD clinical trial.

